# Electronic health record in military healthcare systems: A systematic review

**DOI:** 10.1371/journal.pone.0313641

**Published:** 2025-02-12

**Authors:** Amir Torab-Miandoab, Mahdi Basiri, Arasb Dabbagh-Moghaddam, Leila Gholamhosseini

**Affiliations:** 1 Department of Health Information Technology, School of Paramedical Sciences, AJA University of Medical Sciences, Tehran, Iran; 2 Department of Health Information Technology, School of Management and Medical Informatics, Tabriz University of Medical Sciences, Tabriz, Iran; 3 Department of Knowledge Management, School of Social Science, AJA University of Command and Staff, Tehran, Iran; 4 Department of Public Health and Nutrition, AJA University of Medical Sciences, Tehran, Iran; 5 Infectious Diseases Research Center, AJA University of Medical Sciences, Tehran, Iran; National Research Council, ITALY

## Abstract

**Introduction:**

The immediate access to detailed patient data is vital for effective medical care in military and emergency scenarios, enhancing diagnosis, treatment, and monitoring of military personnel. The integration of electronic health records (EHRs) is urgently needed in military healthcare systems, despite the distinct hurdles involved. Current literature on EHR use in military healthcare is lacking and disjointed. This study aims to bridge this gap through a systematic review, offering a thorough examination of the advantages, obstacles, and recommended strategies for implementing EHRs in military healthcare environments.

**Materials and methods:**

According to the PRISMA guideline, a comprehensive electronic search of all relevant literature on the topic was carried out across multiple databases, including PubMed, Web of Science, Scopus, IEEE, ProQuest, MEDLINE, Cochrane Library, Embase, SID, and ISC up to July 20, 2024. The inclusion criteria involved choosing English-language articles that were available in full text and closely aligned with the study’s objectives. The data extraction sheet for each study included information including the authors, publication year, country, research goals, architecture and components, context, processes involved, standards utilized, platform and technology, level of implementation, interoperability issues, challenges faced, information resources, and significant findings.

**Results:**

A total of 9,618 titles were retrieved from different databases. After removing duplicates, 6,051 titles were left. Upon evaluation, 29 articles were chosen for inclusion in the review. The results show that most of the studies were carried out at the United States Department of Defense (DoD) level with the aim of improving the quality of care and patient safety, as well as integrating healthcare delivery. Additionally, the studies covered various processes such as clinical documentation, appointment scheduling, research, telemedicine, decision support, and computerized physician order entry. Health level seven fast healthcare interoperability resources (HL7 FHIR), clinical document architecture (CDA), health insurance portability and accountability act (HIPAA), international classification of diseases 10th and 9th revision (ICD 10, ICD 9), international organization for standardization technical committees (ISO TC), software development kits (SDKs), and web-based architecture are some of the most important requirements for implementing EHR. The most significant challenges reported in the implementation of EHR included concerns about privacy and security, the sensitive military deployment environment, infrastructure limitations, and interoperability concerns.

**Conclusions:**

Policymakers and practitioners can get insight from the findings regarding the standards that must be met, the challenges that must be overcome, and the requirements for EHR implementation in military healthcare settings. It could be a useful starting point when implementing EHRs in military healthcare systems, especially in nations where e-health development and planning are still in their early stages.

## Introduction

In recent years, the utilization of Electronic Health Records (EHRs) has gained immense popularity and has become a significant component in modern healthcare systems [1]. EHRs have revolutionized the healthcare industry by transforming the way medical information is stored, managed, and shared [2]. EHRs refer to digital versions of patients’ medical records that are stored and accessible through secure electronic platforms and contains medical history, diagnoses, medications, treatment plans, immunization dates, allergies, radiology images, and laboratory test results. These digital systems offer numerous advantages, including improved patient care, enhanced clinical decision-making and streamlined healthcare processes [3].

While the adoption of EHRs is widespread across various healthcare settings, their implementation within military healthcare systems brings unique challenges and opportunities [4]. Military healthcare systems necessitate specialized medical care to cater to the distinct health needs and challenges encountered by military personnel, including combat injuries, mental health issues, and the repercussions of deployment. It is imperative for military healthcare systems to guarantee easy accessibility to healthcare services for national guard and reservist roles, veterans, and their families, irrespective of their location or deployment status. Ensuring continuity of care is essential as military personnel may frequently relocate due to deployments, reassignments, or transitions to civilian life [5].

The upkeep of the health and readiness of national guard and reservist roles is a top priority for military healthcare systems to ensure they are physically and mentally prepared for their duties. These systems must be appropriately equipped to provide trauma care for combat injuries and emergencies during military operations. Comprehensive mental health services must also be offered to address the unique stressors and mental health challenges experienced by military personnel. Emphasis on preventive care is crucial to promote overall health and well-being and prevent injuries and illnesses in the military population. Investment in research and innovation is vital to continually enhance medical treatments and technologies for the benefit of military personnel. Collaboration with civilian healthcare providers may be necessary to ensure seamless care for military personnel and their families, particularly during transitions to civilian life or when stationed in remote areas. Adequate funding, staffing, facilities, and equipment are indispensable for military healthcare systems to effectively meet the diverse healthcare needs of military personnel and their families [6,7].

Military healthcare systems are responsible for providing health and medical care to military personnel, including active-duty service members, veterans, and their dependents. These systems operate in diverse and often complex settings, ranging from military bases to combat zones, and are designed to ensure the health and well-being of military personnel both during peacetime activities and in times of conflict. The provision of high-quality healthcare services is essential for safeguarding the health, well-being, and operational readiness of military forces [5].

The integration of EHRs in military healthcare systems offers the potential to revolutionize medical practices within these specialized contexts. By replacing traditional paper-based records with digital systems, EHRs enable healthcare providers to access comprehensive patient information in real-time, regardless of their location [6]. This accessibility can prove crucial, particularly in military deployments or emergency situations where rapid access to accurate medical data can enable efficient diagnosis, treatment, and monitoring of military personnel and significantly impact patient outcomes [7].

The necessary integrations for a military EHR system include the ability to communicate with other military healthcare systems, robust encryption and authentication measures to protect sensitive patient information, tracking and managing the health status of deployed service members, and modules for monitoring military-specific health concerns. It is important for the military EHR system to be customized to meet the unique needs of military healthcare providers and patients in order to ensure efficient delivery of healthcare services [6].

However, implementing EHRs in military healthcare settings presents unique challenges. These challenges encompass technical considerations, such as deploying and integrating EHR systems across different military branches, ensuring interoperability between military and civilian healthcare providers, and addressing data security and privacy concerns in sensitive military environments [8]. Moreover, the specific requirements of military healthcare, including the need for adaptability to different operational contexts and compatibility with deployed medical facilities, necessitate tailored solutions for successful EHR implementation [9].

To date, the literature on EHR utilization in military healthcare systems remains limited and fragmented. A comprehensive review of existing studies is therefore essential to gain a comprehensive understanding of the impact and effectiveness of EHRs within military healthcare settings. By conducting a systematic review, this manuscript aims to address this gap in knowledge and provide an in-depth analysis of the benefits, challenges, and best practices associated with employing EHRs in military healthcare settings.

The objectives of this systematic review are two-fold: firstly, to evaluate the impact of EHRs on healthcare outcomes, patient safety, and resource management within military healthcare systems; secondly, to identify the key barriers, lessons learned, and best practices for successful EHR implementation in military contexts. By synthesizing the available evidence, this review aims to inform policymakers, healthcare providers, and researchers in optimizing EHR utilization to enhance the quality, efficiency, and effectiveness of healthcare services for military personnel and their dependents. Overall, this manuscript aims to provide a comprehensive understanding of the utilization of Electronic Health Records in military healthcare systems. By examining the unique challenges and opportunities associated with adopting EHRs in these specialized contexts, this systematic review strives to facilitate evidence-based decision-making and facilitate the advancement of healthcare within military settings.

## Materials and methods

This review adhered to the preferred reporting items for systematic reviews and meta-analyses (PRISMA) guidelines, which provide a standardized set of criteria for reporting systematic reviews and meta-analyses. While PRISMA is primarily designed for reviews evaluating intervention effects, it can also be used for studies addressing other objectives such as etiology, prevalence, diagnosis, or prognosis. The goal of PRISMA is to enhance the clarity and quality of reporting in systematic reviews. Additionally, it can be useful for critically appraising existing reviews, though it does not serve as a tool for assessing their overall quality [10].

### Information source

Studies were identified up to July 20, 2024, through a comprehensive search conducted across several online databases including PubMed, Web of Science, Scopus, IEEE, ProQuest, MEDLINE, Cochrane Library, Embase, SID, and ISC. The search encompassed online books, published papers, conference abstracts, seminar and reference publications to minimize publishing bias and ensure the inclusion of a significant number of articles. Moreover, the reference lists of selected articles were examined for additional relevant studies. Additionally, manual searches were conducted in the bibliographies of articles and reviews. To stay updated, an email alert system was set up in the electronic databases, monitoring new publications that met the selection criteria based on the saved search history as of July 20, 2024.

### Eligibility criteria

Two reviewers (A TM and L Gh) independently evaluated the titles of the articles. The articles were divided into two categories: "definitely exclude" and "possibly include." The abstracts of the articles in the "possibly include" category were evaluated. Studies that had abstracts that didn’t meet the inclusion criteria were excluded. The entire text of the remaining articles was examined, and any duplicate articles were removed. Articles were considered eligible if they reported on implementation, development, validation, or translation studies of electronic health records in the context of military healthcare systems. Only articles written in English and with full-text accessibility were included. Letters to the editor, commentary, review, and opinion papers were excluded. There was no restriction on the publication year.

### Search strategy

The search terms were generated based on the ideas or concepts mentioned in the research objective: Electronic Medical Record(s), Electronic Health Record(s), Computerized Medical Record(s), Automated Medical Record(s), Medical Record System(s), Patient Record System, Patient Management System, Armed force, Army, Military, Air Force, Navy and Healthcare. The syntax of search strategy was produced in Table 1. The search strategy was approved by a medical librarian and information specialist. Also, the authors confirm that the search strategy is not pre-registered.

**Table 1:** The syntax of search strategy.

Databases	PubMed, Web of Science, Scopus, IEEE, ProQuest, MEDLINE, Cochrane Library, Embase, SID, and ISC.
Date	up to 2024-7-20
Strategy	#1 AND #2 AND #3
#1	("Electronic Medical Record" OR EMR OR "Electronic Health Record" OR EHR OR "Computerized Medical Record" OR CMR OR "Automated Medical Record" OR AMR OR "Medical Record System" OR "Patient Record System" OR "Patient Management System")
#2	(Army OR Military OR "Armed force" OR "Air Force" OR Navy)
#3	Healthcare

Initially, articles that might be relevant were chosen primarily based on their titles and imported into Endnote. Subsequently, a more refined selection process was conducted, considering the abstracts and full texts of the articles.

### Data collection process

The reviewers (A TM, M B, A DM and L Gh) independently reviewed the full text of the selected articles. A standardized data extraction form was utilized to extract relevant information from these articles. The data extraction sheet for each study encompassed details including authors, year, country, research objective, architecture and components, setting, included processes, standards used, platform and technology, extent of implementation, interoperability aspect, challenges, information resource, and key findings. The results from all the selected studies were gathered and compiled. To ensure accuracy and completeness of the data entries, another independent investigator reviewed and verified them. There was no missing data in the present study.

### Quality assessment

To support the inclusion/exclusion process, an assessment and analysis were conducted to evaluate the quality of the initially selected studies. The critical appraisal tool used for this purpose was the Quality Assessment of Diagnostic Accuracy Studies (QUADAS) checklist. This tool is specifically designed to assess the reliability and validity of combined studies or to evaluate validity and reliability as separate components [11].

The checklist consists of 14 questions, each with three options: yes, no, and unclear. Two researchers thoroughly evaluated all the identified studies for potential bias. During the assessment, if a study provided information corresponding to each checklist question, it was scored as "yes." If the study did not clearly report the required information, the item was scored as "no." In cases where the information provided was incomplete, the item was scored as "unclear." Based on previous research, a score of 7 out of 14 or higher, with the majority of items scored as "yes," indicated a high-quality study, while scores below 7 indicated a low-quality study. All discrepancies between the researchers’ assessments were resolved through agreement.

To present the results, various software tools were utilized, including Excel, XMind, an online word cloud generator, and the yED graph editor.

## Results

**Fig 1:**
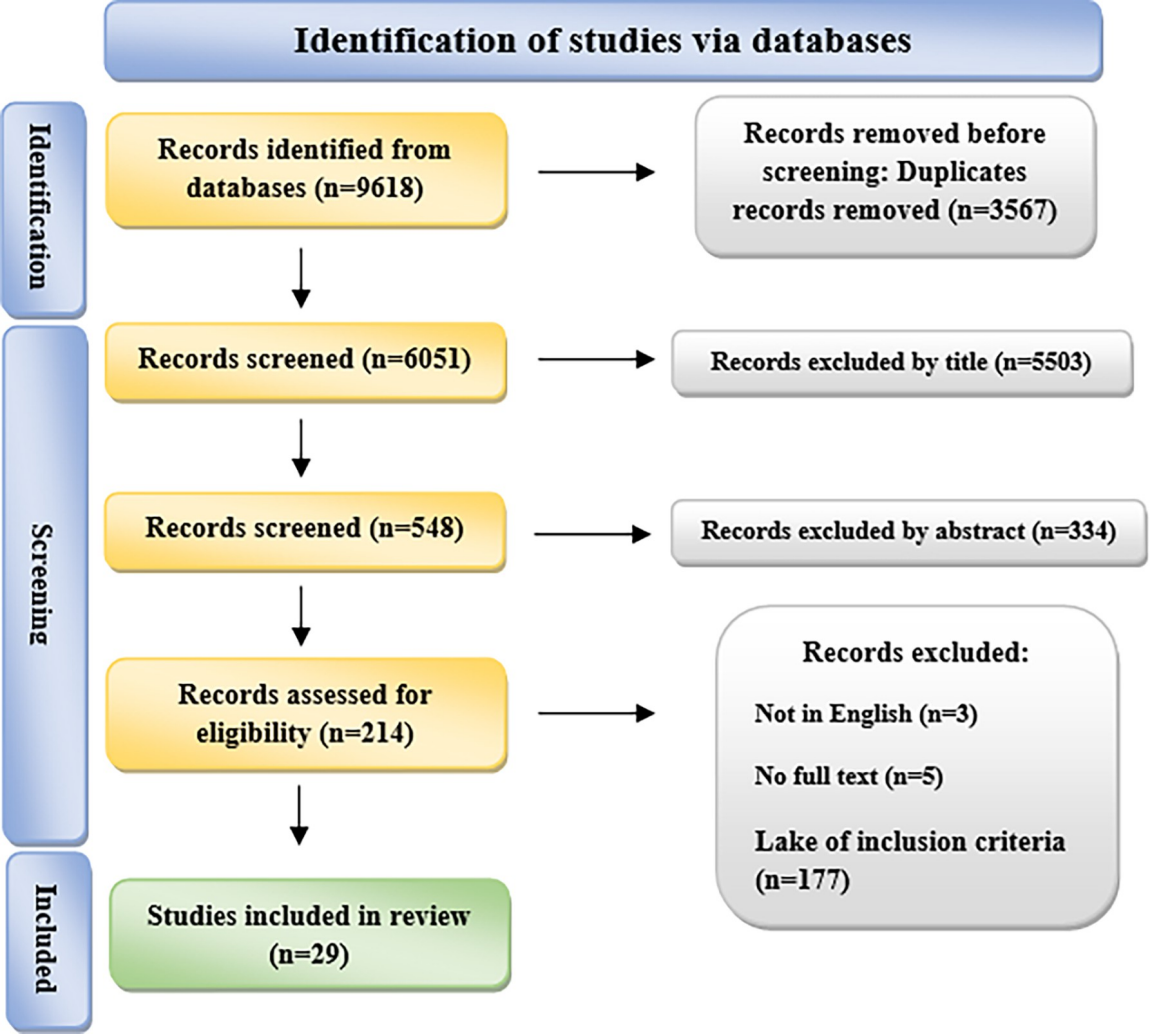
PRISMA flow diagram.

**Fig 2:**
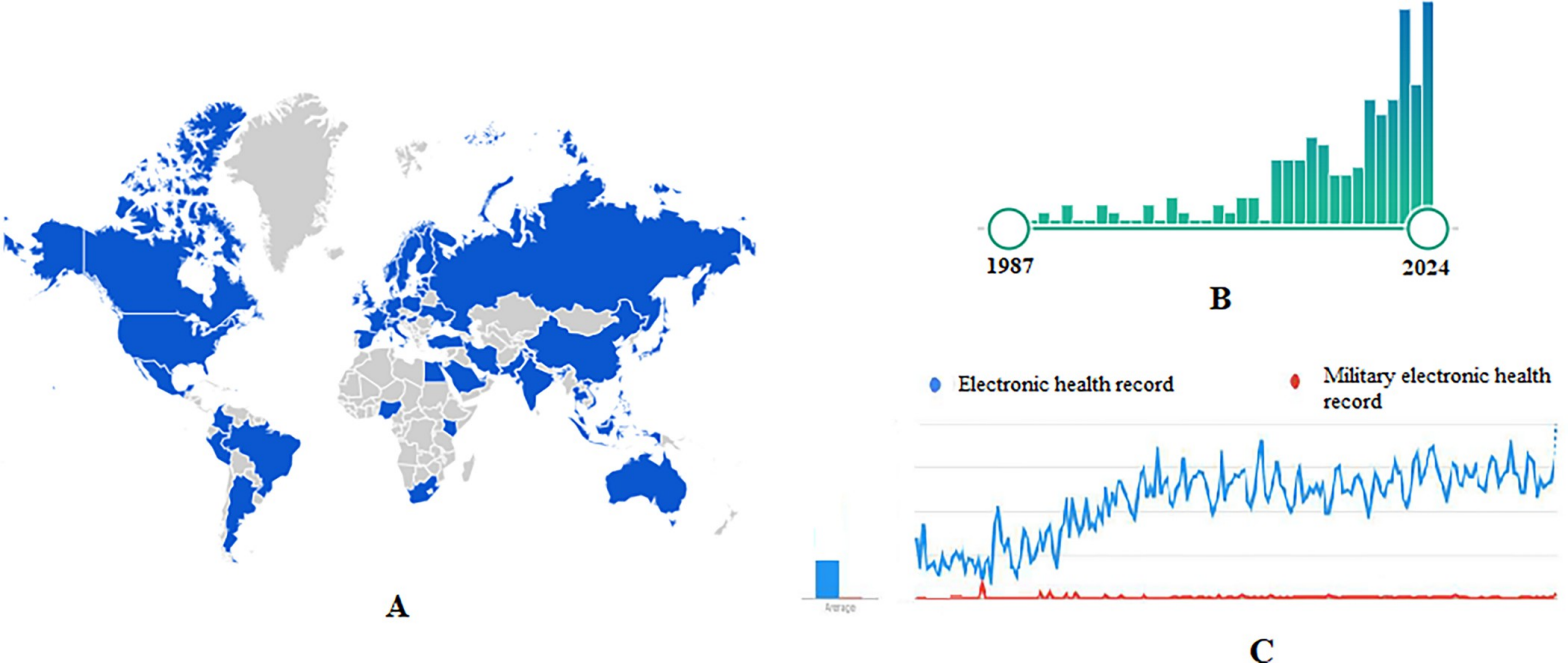
(A) Geographical scope in the subject of electronic health record in the world based on the google search (B) The growth trend of articles in the field of military electronic health record in databases (C) The comparison of the google search trend of electronic health record and military electronic health record.

Fig 1 illustrates a flow diagram that follows the PRISMA guidelines, providing a detailed overview of the article selection process. Initially, a total of 9618 titles were obtained from various databases. After eliminating duplicate articles, 6051 titles remained. Two reviewers carefully evaluated the titles for relevance, resulting in the exclusion of 5503 articles that were deemed irrelevant to the review topic. Out of the remaining titles, 548 articles were selected based on their titles. The abstracts of these articles were then analyzed, leading to the exclusion of 334 articles that did not meet the eligibility criteria. As a next step, a total of 214 full-text articles were retrieved and assessed against the eligibility criteria. Following the assessment, 185 articles were excluded (please refer to Fig 1 for further details), and ultimately, 29 articles were included in the review. Fig 2 shows global trends in electronic health records.

The results indicate that the majority of the studies were conducted at the Department of Defense level, with the goal of cost savings, increased user satisfaction, improved care, patient safety and communications, and the transition to a modern, value-based managed care system and integrated healthcare delivery. Other objectives included the reducing medical errors, exchange of health information, improved access to information, and maintaining confidentiality and security of information. Additionally, the studies aimed to improve the quality, quantity, and completeness of information, empower patients and treatment staff, and promote clinical research. Most of the studies were conducted in hospitals and clinics within the United States.

**Fig 3:**
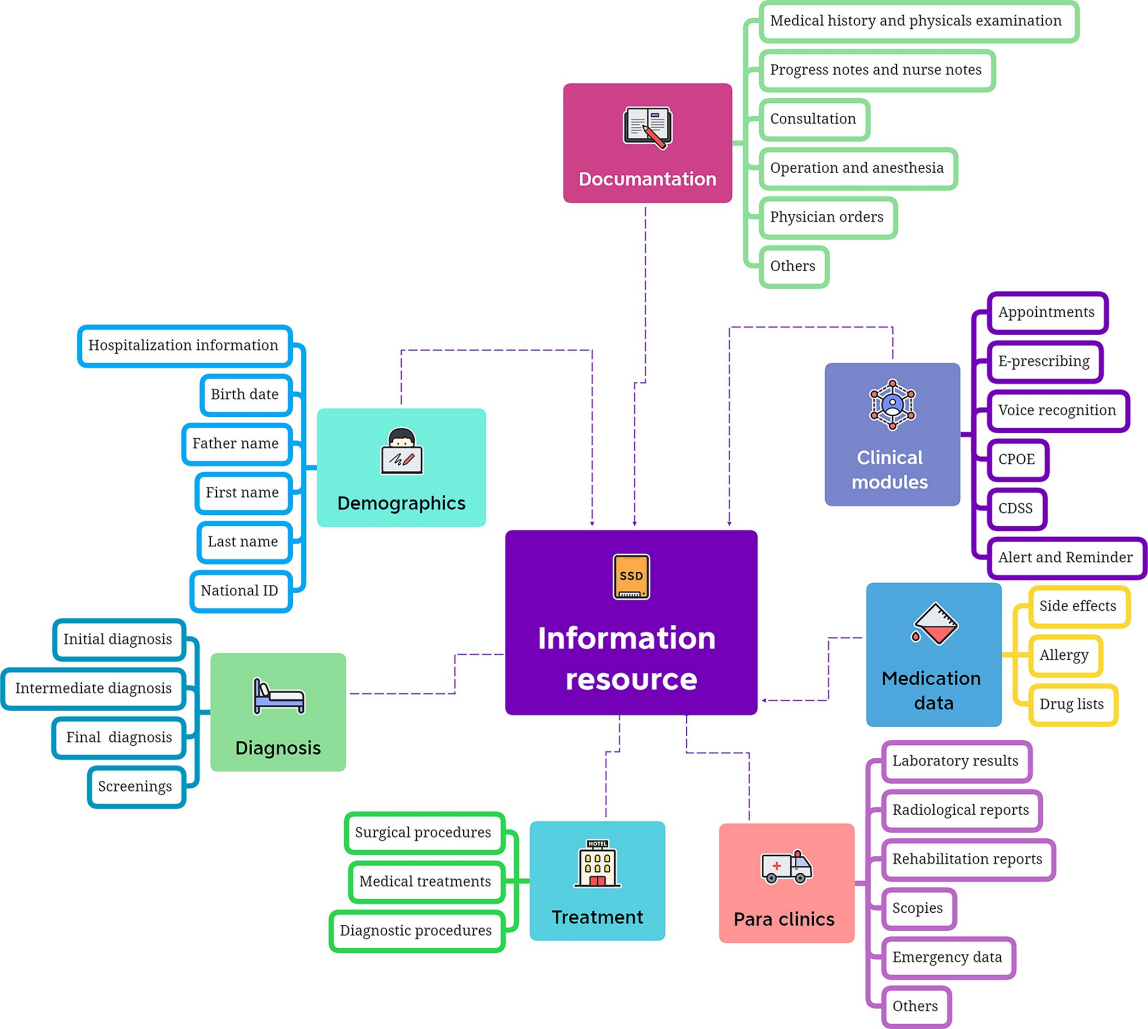
Summary of EHR information resources components based on findings.

Furthermore, the studies reported on various processes such as clinical documentation, appointment setting, e-prescribing, querying, data mapping, disease surveillance, telemedicine, decision support, computerized physician order entry (CPOE), and information management. The interoperability between systems was predominantly at the semantic level and relied on the Military Health System as the primary information source. Fig 3 summarizes the different components of information resources in the EHR that are mentioned in studies.

The most commonly used transport standard was health level seven fast healthcare interoperability resources (HL7 FHIR), the most frequently used content standard was clinical document architecture (CDA), and the most commonly used terminology standards were international classification of diseases ninth revision (ICD 9) and international classification of diseases tenth revision (ICD 10). Additionally, the most commonly used security standards were health insurance portability and accountability act (HIPAA), and the most frequently used technical standards were international organization for standardization technical committees (ISO TC). In the majority of the studies, software development kits (SDKs) were utilized as development platforms for electronic health records. Among modern architectural approaches, web-based architecture was the most commonly used.

The most significant challenges reported in the implementation of EHR included resistance to change, privacy and security concerns, a wide range of architectural options and capabilities, unclear support policies and procedures, the sensitive military application environment, infrastructure limitations, user training difficulties, time and financial costs, challenges in creating scalability and reusability, a variety of health data types and their large volume, as well as concerns about interoperability. Details of the selected studies in this study are given in Table 2.

**Table 2:** Details of the selected studies.

Authors and Year	Country	Research objective	Challenges	Architecture and components	Setting	Included processes	Used standards	Platform and technology	Extent of implementation	Interoperability aspect	Information resource	Key findings	QUADAS score and risk of bias
Bar-Dayan Y, et al. 2013 [12]	Israel	Use of EHR to cost saving.	It was difficult to make changes.	Web-based	All specialty clinics	Clinical documentation, management of referral system, appointment setting, e-prescribing, exchange of information, and submission of quality measures to CMS	HL7 FHIR	Computer, windows form generator	Israeli Defense Forces	Semantic	EHR meaningful use 1. Clinical documentation: patient demographic, medication lists, physicians’ notes (eg, medical history and follow-up), problem lists 2. Test and imaging results: laboratory reports, radiological reports 3. Computerized provider order entry: consultation requests, e-prescribing 4. Decision support: clinical guidelines, drug-allergy alerts, drug-dose support	EHR can facilitate effective utilization of healthcare providers and decrease costs.	9 (low risk)
Do NV, et al. 2011 [13]	United States	Design, build, implement, and evaluate a PHR, tethered to the Military Health System based on user preference.	Regardless of model design, implementation challenges were formidable given barriers and debate on key issues such as privacy and security, architecture options, functionalities, and supporting policies.	Service-oriented architecture	Hospitals, clinics	Clinical documentation, exchange of information, query, data mapping	CCD, CCR, HIPAA, HL7, CDA	Google Health, Microsoft HealthVault, laptop, server and sql server, SOAP, API, SDK, Microsoft.NET, XML, HTML	Madigan Army Medical Centers	Semantic	Military Health System 1. Laboratory results 2. Allergies 3. Medications 4. Radiology reports 5. Appointments 6. Medical procedures 7. Medical problems list 8. Consultation reports 9. Inpatient notes 10.Outpatient encounter notes	PHR is a powerful tool for patient activation. Adopting standards into design can enhance the national goal of portability and interoperability.	10 (low risk)
Spott MA, et al. 2018 [14]	United States	Use of EHR for improvement of trauma care.	Typically, the military capture of the medical record in a deployed environment is challenging.	Web-based	Hospitals	Clinical documentation, communication between healthcare providers, research	ICD 9/10, HIPAA	SDK	Department of Defense	Structural	Military Health System 1. demographics 2.treatments 3.outcomes 4. injury	By employing high data acquisition standards and automated identification issues, the EHR offers an increased chance to trend information and improve patient care.	8 (low risk)
Khan S. 2018 [15]	United States	Shift toward a modern value-based managed care system, which requires much more integrated healthcare delivery.	Building infrastructure, unified clinical leadership and health system reform are challenging.	Service-oriented architecture	Hospitals, clinics	Exchange of information, data analytics	HL7 FHIR	SDK	Department of Defense	Semantic	Military Health System 1. demographics 2. drug prescriptions 3. diagnoses 4.treatment 5. paraclinic	Key outcomes from this effort include health quality improvement based on national standards, greater efficiency with reduced care variation and medical error prevention, improved population health outcomes and early disease detection, and prevention and treatment activities among others.	11 (low risk)
Hoyt R, Yoshihashi A. 2010 [16]	United States	Implementation of voice recognition for documenting outpatient encounters in the EHR.	Training, inaccuracies, missing error and time wasted correcting notes are challenging.	Web-based	Hospitals, clinics	Clinical documentation	SNOMED -CT, AHIMA	Voice recognition software (Dragon NaturallySpeaking), computer hardware (RAM, CPU, headset microphone), mobile, wireless settings	Department of Defense	Technical	EHR meaningful use 1.clinical elements	Software was accurate, was faster than typing, improved note quality, and permitted closing a patient encounter the same day.	11 (low risk)
Chretien JP, et al. 2016 [17]	United States	Health information exchange for study the long term health consequences of military services.	Securing participation from separating service members, need for political will to implement the program and establishing ethically appropriate and technically feasible procedures for obtaining informed consent to participate are challenging.	Web-based	All healthcare centers	Exchange of information, disease surveillance, research	HL7 FHIR	Internet, computer, network settings	Department of Defense	Semantic	Military service records 1.demographic and occupational characteristics 2.immunizations 3.clinical medical encounters (outpatient and inpatient) 4.laboratory testing 5.deployment, pre- and post-deployment health assessments 6.reportable medical event reports 7.casualties of all service members	Knowledge of the health effects of military service improves; the health information exchange infrastructure could accelerate application of this knowledge to clinical practice at the point of care.	8 (low risk)
Giannakopoulou O, et al. 2021 [18]	Greece	Digitalize the medical examination procedure of recruitment phase of conscripts in the Hellenic Navy.	The challenge is that the delay in process is not decreased; however this is done for the benefit of the quality which increases significantly.	Web-based	Military medical units	Telemedicine, screening, advising, decision making, diagnosing, prediction, medical data collection and recording	Advanced Encryption Standard	Digital tools, database, machine learning, medical devices, computer, API, Barcode, bluetooth, printer	Hellenic Navy	Technical	National health care association 1. pre-screening test (cardiovascular risk data) 2. demographics 3. personal and family medical history	eMass solution contributes to beneficial management and medical data analysis, preventing inessential physical or medical examinations minimizing danger of possible errors and reducing time-consuming processes. Moreover, eMass exploits Electronic Health Record data through a machine learning based cardiovascular risk assessment tool.	12 (low risk)
Jingquan L. 2017 [19]	United States	Present a SOA-based approach for the integration of EHR systems and PHR systems and identify the primary focus areas for interoperable and secure PHR systems.	Interoperability, reusability, and scalability along with security and privacy are challenging.	Service-oriented architecture	Hospitals, clinics	Collect, store, use, share of information, computerized physician order entry, clinical decision support	HL7, CDA, HIPAA, HITECH, IHE XDS	Patients Like Me, 23 and Me, Microsoft HealthVault, Google genomics for personal health information management, wearable devices, computers, smart phones, mHealth devices, inter-organizational service bus, SOAP, REST, web services, Oracle, Vista, WSDL	Department of Defense	Semantic	Military Health System 1. medical records 2. lab and radiology results 3. medications 4. self-entered health information	PHR will empower individuals to manage their own health care, reduce medical errors, improve communications with doctors, lower health care costs, and provide vital and complete information for emergency care.	13 (low risk)
Singla M, et al. 2020 [20]	United States	Use of ICD-9-CM codes in EHR to increase diagnostic accuracy in inflammatory bowel disease.	Using "practical" or "retrospective" diagnoses rather than confirmed diagnoses is challenging.	Web-based	Hospitals	Research, clinical documentation	ICD-9-CM, ICD 10	SDK	Department of Defense	Semantic	Military Health System 1.procedures 2. diagnosis 3. demographic 4. medical history 5. medications 6.colonoscopies, radiological studies and lab test	The results showed that poor codes for inflammatory bowel disease had improved greatly.	9 (low risk)
Sloane E, et al. 2007 [21]	United States	Development of EHR to support decisions and to improve the quality, quantity, and completeness of information available to physicians.	Healthcare depends on multiple disparate care-delivery-providers, each using specialized computer systems for optimal clinical data and practice management, which is challenging.	Service-oriented architecture	Hospital	Exchange of information	HIPAA, HL7 FHIR	XML	Department of Defense	Semantic	National Healthcare Information Network 1. demographics 2. drug prescriptions 3. diagnoses 4.treatment 5. paraclinic	These features could be used to improve individual and aggregate static data exchange between healthcare centers and improve a patient’s care.	10 (low risk)
Friedman DR, et al. 2022 [22]	United States	Integration patient-reported outcome data into EHR to bring together disparate sources of patient information and improve medical care.	Integration of patient-reported outcome is challenging, because requiring consideration of heterogeneity of data sources, patient population, data display, provider education, and guidelines and pathways.	Web-based	hematology-oncology clinics	Documenting of cancer-related data, exchange of information	ICD-9-CM, ICD-10	Registry system	Veteran administration medical centers	Semantic	Veteran Symptom Assessment System 1. Patient demographic 2.laboratory data 3.radiologic data 4. disease 5. symptom and sign	With this method, it is possible to identify patients who are experiencing severe, long-term or multiple symptoms in real time and provide immediate intervention and improve the quality of life.	11 (low risk)
Smith B, et al. 2008 [23]	United States	Self-reporting of medical conditions with EMR.	Patient-clinician communication, multiple data sources and health literacy of the patient are challenging.	Web-based	Hospitals, clinics	Documenting of medical-related data, exchange of information	ICD-9-CM	Registry system	Department of Defense	Semantic	Military Health System 1. Patient demographic 2. medical conditions 3.diagnosis 4. medical treatment	Data integrity increased within the medical record information with this approach.	12 (low risk)
Li L, et al. 2014 [24]	China	Improvement of electronic health records and health promoting.	There are problems, such as optimization of system structure, perfection of business function, unity of standard and system.	Web-based	All healthcare centers	Decision support, data collection, summarizing, statistics, demonstration, exchange of information	ISO/TS 18308: 2004	Satellite communication network, wired and wireless network, Oracle data base, digital signature, server	Ministry of health of China	Technical	Institute of Health Service and Medical Information 1. health information 2. data dictionary 3. patient demographic 4. medical treatment service	Construction of this system achieves various health data integration, completes health evaluation criterion, enforces standard health management process, and makes full use of information and health resource.	9 (low risk)
Wong KH, et al. 2020 [25]	United States	Accurate documentation of injuries and treatments to improvement of care, both in the immediate post injury phase and the longer-term recovery.	Support different equipment’s, low communication bandwidth and future expansions are challenging.	Web-based	All healthcare centers	Documenting of medical-related data, telemedicine, exchange of information	ICD 10	Sensors, wireless communication, dedicated keyboard, bar code scanner, cables, mobile, Bluetooth, audio and imaging hardware,	Department of Defense	Semantic	Military Health System 1.demographics 2.medical data 3. paraclinics 4. references 5. share	The widespread deployment of this type of device will enable more effective health care, limit the impact of battlefield injuries, and save lives.	11 (low risk)
Blosnich JR, et al. 2020 [26]	United States	Determination of the prevalence of social determinants of health documentation in the EHR and how social determinants of health are associated with suicide ideation and attempt.	Interagency data sharing to enrich health services research is challenging.	Web-based	All healthcare centers	Documenting of medical-related data, exchange of information	ICD 10	Network settings	Department of Defense	Semantic	Veterans Health Administration 1. sociodemographic 2. mental health data	Integration of social determinants of health data in EHR could improve suicide prevention.	12 (low risk)
Thériault FL, et al. 2022 [27]	Canada	Use of Electronic Medical Record data to identify new diagnoses of major depression and spinal pain.	This study was limited to pain and depression models, which is a challenge and comprehensive models are needed.	Web-based	Hospitals	Documenting of medical-related data, exchange of information	ICD 10, DSM-5	SDKs	Canadian Armed Forces	Semantic	1.demographics 2. mental health data 3. brain and nerve problems data	Results highlight the importance of correcting for misclassification in electronic medical record data research.	13 (low risk)
Rockswold PD and Finnell VW. 2010 [28]	United States	Use of predictors’ tools to upgrade the system and increase user satisfaction.	Slow screen refresh rate, computer literacy, lack of buy-in from end users, effects on clinical staff efficiency and effects on clinician–patient communication cited as problems.	Web-based	Hospitals and clinics	Documenting of medical-related data	ICD 10	Templates, tablet, wireless networks, computer	Department of Defense	Structural	Military Health System 1.demographics 2. order set 3. diagnosis	Training, duration of usage, and provider role are associated with use of advanced features.	14 (low risk)
Silva R, et al. 2017 [29]	Ecuador	Development of compatible approach to interoperate EHR.	Interoperability issues are challenging.	Service Oriented Architecture	Hospitals and clinics	Documenting of medical-related data, exchange of information	ISO 13606, SNOMED CT, ISO TC 215, ICD-10	PostgreSQL, hibernate, spring, java script, XML, API, WSDL, SOAP, Link EHR ED, Free Mind, HTML	Ministry of Public Health	Semantic	ISO Archetype 1.demographics 2. clinical data 3. emergency data 4. paraclinic data	The present approach will contribute to promote the interoperability of the medical records implemented in Ecuador, which will improve the quality of health care.	12 (low risk)
Wilk JE, et al. 2016 [30]	United States	Recording diagnoses and treatment factors related to post-traumatic stress disorder in the EHR.	Not recording was challenging due to stigma reduction or career protection.	Web-based	Clinics	Documenting of medical-related data	DSM-5, ICD 9	SDKs	Department of Defense	Technical	Military Health System 1.demographics 2. treatment 3. diagnosis	Despite the benefits of recording information in EHR, some doctors still do not record information in it.	9 (low risk)
Reimer RJ, et al. 2023 [31]	United States	Assembling an EHR dataset for a large cohort of military Veterans diagnosed with amyotrophic lateral sclerosis.	Managing huge amounts of data is difficult.	Web-based	Hospitals	Documenting of medical-related data	ICD 9, ICD 10	Python library	Department of Defense	Semantic	Veterans Health Administration 1. demographics 2. diagnoses 3.vital signs 4.paraclinic values 5.prescriptions 6.procedures	EHR data are an invaluable source for clinical information and can be useful for understanding diseases and their treatments when analyzed appropriately.	12 (low risk)
Ladan A and Daura UD. 2015 [32]	Nigeria	Creating and maintaining and managing patients’ medical records in electronic form	Lack of Information and communications technology facilities, absent of finding aids, inadequate space and storage facilities are the challenges.	Web-based	Hospitals	Documenting and managements of medical-related data	ISO 13606	SOAP	Nigeria military hospitals	Technical	1. demographics 2. diagnoses 3. patient’s complaints 4.paraclinic values 5.procedures and treatments	The use of EMR leads to overcoming the challenges of traditional methods of records management.	11 (low risk)
Herzog CM, et al. 2015 [33]	United States	Use of EHR to determine the prevalence of Metabolic Syndrome; look for trends in prevalence over time compare prevalence between.	In this study, only the use of EHR in the prevalence of metabolic syndrome for active duty is reported.	Web-based	Hospitals and clinics	Documenting of medical-related data, exchange of information	ICD 10	SDKs	Department of Defense	Semantic	Military Health System 1. demographics 2. diagnoses 4.paraclinic and vital values 5.procedures and treatments	The use of EHRs can explore policies to help reduce outbreaks.	10 (low risk)
Leightley D, et al. 2018 [34]	England, Scotland, Wales	Development of integrated EHR to capture summaries of care and contact made to healthcare services as well as produce a multi-nation dataset of secondary health care.	With outpatient care being sparsely coded making it challenging for use in epidemiological research and matching process due to complex structures is challenging.	Web-based	Hospitals	Documenting of medical-related data, exchange of information	ICD 10, OPCS, HL7 FHIR	SDKs	England, Scotland and Wales Armed Forces	Semantic	National Health Service 1. demographics 2. drug prescriptions 3. diagnoses 4.treatment 5. paraclinic and vital values 6. clinical notes 7. patient self-reported illness	EHR was designed to monitor the cost of treatment, enable administrative oversight, and epidemiological research.	9 (low risk)
Bramoweth AD, et al. 2018 [35]	United States	Examination insomnia and insomnia-related care with EHR data.	Despite efforts to standardize aspects of data entry in the EHR, variation and inaccuracy persisted.	Web-based	Hospitals and clinics	Documenting of medical-related data, exchange of information	ICD 9, ICD 10	Data warehouse,	Department of Defense	Semantic	Office of Mental Health Services 1. sociodemographic 2.diagnoses 3.medications 4. assessment and treatment	EHR can help to improve care and improve veteran health and quality of life.	12 (low risk)
Leightley D, et al. 2023 [36]	England	Development a tool for indicating veteran status across all healthcare services.	This system does not rely on any coding structure or predefined fields and solely uses free-text documents.	Web-based	Hospitals and clinics	Documenting of medical-related data, exchange of information, determining the healthcare needs of veterans	N/A	Rule-based approach, SQL, machine learning, NLP, python	England Armed Forces	Semantic	National Health Service free text style 1. demographics 2. drug prescriptions 3. diagnoses 4.treatment 5. paraclinic	EHR has the potential to be used to support veterans for clinical documentation purposes.	11 (low risk)
Milenković D, et al. 2012 [37]	Serbia	Building a comprehensive and integrated EHR, which will enable the collection and management of all data relevant to a complex healthcare system?	Limited resources and infrastructure were the most important challenges	Web-based	Hospitals and clinics	Documenting of medical-related data, exchange of information, information management	HL7, CDA	ICT equipment	Ministry of Defense of the Republic of Serbia	Semantic	1.scheduling of examinations 2.diagnosis 3.paraclinics 4.pharmacy 5.procedure	The main features of the new approach are orientation to a patient, health care based on evidence, exchange of information about the health of a patient in order to improve health services and reduce costs.	10 (low risk)
Charles MJ, et al. 2005 [38]	United States	Use of EHR to automate patient data documentation, leading to improvements in patient safety.	The main challenge of EHR is ensuring the security and privacy of sensitive patient information.	Web-based	Hospitals and clinics	Documenting of medical-related data, exchange of information, information management, CPOE	HL7, CDA	Information systems, CDR	Department of Defense	Semantic	Military Health System 1. demographics 2. drug prescriptions 3. diagnoses 4.treatment 5. paraclinic and vital values	Secure online access to longitudinal health records is possible using EHR that greatly improving overall health care delivery and supporting patient safety initiatives for beneficiaries.	9 (low risk)
Lucas JE, et al. 2017 [39]	United States	Use of EHR data to build a model for treatment of cardiovascular disease.	EHR are an increasingly common source of data that are challenging to analyze.	Web-based	Hospitals and clinics	Documenting of medical-related data, exchange of information, information management, research	ICD 10	SDKs	Department of Defense	Semantic	Military Health System 1. demographics 2. drug prescriptions 3. diagnoses 4.treatment 5. paraclinic	Electronic health records data can be used to build a predictive model of treatment adherence that also correlates with cardiovascular benefits.	8 (low risk)
Li R, et al. 2023 [40]	United States	Use of EHR data with machine learning approaches to determination of signs and symptoms of Alzheimer’s disease and prediction it onset earlier.	This study focused on screening for Alzheimer’s disease in the general population, and did not specifically address other disease.	Web-based	Hospitals and clinics	Documenting of medical-related data, exchange of information, research	ICD 10 CM	Machine learning including logistic regression, support vector machine, AdaBoost, random forests	Department of Veterans Affairs, Veterans Health Administration	Semantic	1. demographics 2. drug prescriptions 3. diagnoses 4.treatment 5. paraclinic	This approach may be useful to help identify people at future risk for Alzheimer’s disease.	11 (low risk)

## Discussion

In recent years, the EHR has experienced significant growth, capturing the interest of researchers globally. A comparison between the general EHR topic and the military-specific EHR topic reveals that the scope of the former far surpasses that of the latter. However, findings from the study indicate that the subject of EHR in the armed forces has been a topic of discussion since 1987. Presently, it remains an area of keen interest for researchers (refer to Fig 2). In addition, the use of EHR has also grown significantly in the world in recent years. There are several reasons for this trend:

1. Improved Data Management: EHRs allow healthcare providers to store, retrieve, and share patient data efficiently. This leads to better coordination of care and enhances the overall quality of healthcare services.
2. Accessibility: EHRs enable healthcare professionals to access patient information across different healthcare settings, facilitating continuity of care. Accessibility ensures seamless data exchange between systems, promoting better collaboration among healthcare providers.
3. Research Opportunities: EHRs provide researchers with an abundance of comprehensive and structured data for study and analysis. This wealth of information can help identify trends, patterns, and potential insights into improving healthcare outcomes and treatment effectiveness.
4. Cost Reduction: Electronic health records can potentially reduce healthcare costs by minimizing paperwork, streamlining workflows, and mitigating medical errors. This is especially beneficial in large healthcare systems where efficiency and cost-effectiveness are crucial.
5. Technological Advancements: With advancements in technology, EHR systems have become more sophisticated, incorporating features such as machine learning, natural language processing, and predictive analytics. These capabilities open up avenues for innovation and further research.

Overall, the growth of electronic health records has attracted global interest due to their potential to transform healthcare delivery, enhance research capabilities, and improve patient outcomes [41–43].

To achieve these goals, EHRs must combine different processes, including clinical documentation, appointment setting, e-prescribing, querying, data mapping, disease surveillance, telemedicine, decision support, CPOE, information management and so on for different functions [44].

Military healthcare systems have unique characteristics and needs that require specialized EHR solutions. The environment of military contracts may be less inclined towards the use of EHR and digitalization in general due to several factors:

1. Legacy Systems: The military often operates using legacy systems that have been in place for many years. These systems may be outdated or lack interoperability with modern digital solutions. Upgrading or replacing these systems to accommodate electronic records and digitalization can be complex, time-consuming, and expensive.
2. Security Concerns: The military places a strong emphasis on security due to the sensitive and confidential nature of military operations. There may be concerns about the security of electronic records and potential vulnerabilities of digital systems. Ensuring the integrity, confidentiality, and availability of sensitive military healthcare data is of utmost importance, and transitioning to electronic records requires robust security measures and technologies.
3. Unique Operational Environments: Military operations often take place in remote or harsh environments where infrastructure, including reliable network connectivity and power supply, may be limited. These challenging operational environments may pose obstacles to the successful implementation and maintenance of electronic records systems, making paper documentation more practical and reliable in some situations.
4. Interoperability Challenges: Achieving interoperability between different military systems and electronic health record systems can be complex. The military has highly specialized systems and operational requirements, and integrating the diverse range of systems used by various military branches and agencies may present challenges in terms of data standardization and interoperability.
5. Procurement Process: The procurement processes in military contracts can be lengthy and bureaucratic, involving strict regulations and requirements. These processes may not be well-suited for the rapid adoption and implementation of new technologies like electronic health records and digitalization.

The military needs to ensure that any technology adopted meets its stringent quality standards and is cost-effective in the long run. Military EHRs, while still subject to general healthcare regulations, must also adhere to additional regulations specific to the armed forces. However, it’s worth noting that the military has recognized the potential benefits of electronic health records and digitalization in improving healthcare delivery for military personnel, streamlining processes, and enhancing interoperability among various military systems. Efforts are underway to gradually adopt and integrate electronic records systems within the military healthcare system, while addressing the unique challenges and requirements of the military environment [45–47].

The majority of studies on the use of electronic records and digitalization in military contracts being conducted at the Department of Defense (DoD) level can be attributed to a few factors. The DoD has significant resources, both in terms of funding and personnel, to conduct research and studies. This allows them to invest in exploring the potential benefits and challenges of implementing electronic records and digitalization in military contracts. The DoD is one of the largest organizations in the world, with a vast network of military personnel, healthcare facilities, and support systems. Studying the implementation of electronic records and digitalization within such a large and complex organization can provide valuable insights and help establish best practices that can be applied to other military contexts. As mentioned earlier, the military healthcare system deals with sensitive and confidential information. The DoD is heavily invested in ensuring the security and privacy of this information, making it particularly concerned with studying the impacts and risks associated with electronic records and digitalization [48].

As for the majority of these studies being conducted in the United States, there are a few reasons. The United States, as one of the world’s leading military powers, allocates significant resources to defense-related research. This includes research on topics like electronic records and digitalization within military contracts. The United States has a well-established and extensive military infrastructure, which includes research institutions, military hospitals, and healthcare systems. This infrastructure provides a conducive environment for conducting studies and research on military-specific topics. The United States has a strong network of collaborations and partnerships between academia, industry, and the government. This allows for multidisciplinary research efforts and knowledge exchange, leading to many studies being conducted in the United States.

While the majority of studies have been conducted in the United States, there are also research efforts and studies taking place in other countries that are exploring electronic records and digitalization in the military context. The findings from these studies contribute to the collective understanding of the benefits, challenges, and best practices associated with implementing electronic records and digitalization in military contracts on a global scale [49].

HL7 FHIR, CDA, HIPAA, ICD-10, ICD-9, ISO TC, SDKs and web-based architecture are all important components in the world of EHR.

HL7 FHIR is important for interoperability, modular approach, RESTful APIs, and data standardization. These features enable seamless data exchange, targeted data retrieval, real-time access, streamlined workflow integration, and consistent data representation across different healthcare systems and platforms [50].

CDA is an HL7 standard that defines the structure and semantics for clinical documents. It is widely used in EHR systems for capturing, storing, and exchanging clinical information. CDA enables the structured representation of patient data, improving data accuracy, comprehensibility, and clinical decision-making. It supports clinical document types like discharge summaries, progress notes, and lab reports [51].

In recent years, HL7 FHIR has been increasingly utilized for representing clinical data through the use of "resources", which can then be exchanged via standardized REST APIs. Therefore, FHIR resources are frequently chosen over the HL7 CDA standard due to their lightweight, flexible design, and focus on interoperability in modern healthcare systems. One of the primary reasons for favoring FHIR over HL7 CDA is its ease of implementation and integration with existing systems. FHIR resources leverage modern web technologies such as RESTful APIs, JSON, and XML, which simplifies the work for developers. In contrast, HL7 CDA relies on a complex XML format that may pose challenges in parsing and integration with other systems. Additionally, FHIR resources are modular and granular, enabling more targeted data exchange compared to the monolithic nature of HL7 CDA documents. This granularity facilitates precise data retrieval and exchange between systems, reducing the risk of information overload and enhancing efficiency. Furthermore, FHIR resources are designed to be scalable and adaptable to meet evolving healthcare needs. By allowing for the easy addition of new resources and extensions, FHIR can cater to changing requirements in healthcare systems without requiring significant rework or redesign. In conclusion, FHIR resources are often preferred over the HL7 CDA standard due to their modern design, ease of implementation, scalability, and flexibility in interoperability with other systems [50,51].

HIPAA is a legislation that ensures the privacy and security of patients’ health information. It sets standards for electronic transactions, privacy rules, security measures, and breach notification requirements. Compliance with HIPAA safeguards patient confidentiality, prevents unauthorized access or disclosure of health data, and establishes trust in the healthcare system [52].

ICD-10 and ICD-9 are classification systems used for coding and categorizing diseases, conditions, and procedures in healthcare. ICD-10 is the more recent version and provides more detailed and specific codes compared to ICD-9. These coding systems allow EHR systems to accurately capture and communicate clinical information, support billing processes, facilitate research and analysis, and contribute to interoperability [53].

ISO TC is a technical committee of the International Organization for Standardization that develops international standards for various industries, including healthcare. Standards developed by ISO TC ensure interoperability, quality, and safety in healthcare systems, devices, and informatics. Adherence to ISO standards in EHR systems promotes global compatibility, enhances data exchange, and facilitates collaboration [54].

SDKs provide developers with a set of tools, libraries, and documentation to build applications for a specific platform or framework. In the context of EHR, SDKs can simplify the development process, accelerate application creation, and provide access to APIs and resources for integrating with EHR systems. SDKs enhance developer productivity and promote innovation in the healthcare software ecosystem [55].

Web-based architecture refers to the use of web technologies for designing and delivering EHR systems. This architecture enables access to EHR applications through web browsers, making them platform-independent and easily accessible from different devices. Web-based EHR systems can promote remote access, telemedicine, and patient engagement. They also facilitate updates and maintenance as changes can be deployed centrally without requiring client-side installations [56].

A system-level approach to understanding web-based systems involves looking at the various components and interactions within the system as a whole. In the context of a web-based system, this includes the software application, hardware infrastructure, network connectivity, data storage, security measures, and user interfaces [57]. Some benefits of using a web-based system from a system-level approach include:

- Centralized data: Web-based systems typically centralize data storage, making it easier to manage and access information from anywhere. This facilitates better integration between different modules or components within the system.
- Scalability: Web-based systems can easily scale up or down based on the requirements of the organization. This scalability allows for the system to handle increased traffic, users, or data without major disruptions.
- Interoperability: Web-based systems can provide seamless integration with other systems or applications through APIs (Application Programming Interfaces). This enables data sharing and communication between different systems, improving efficiency and productivity.
- Redundancy and backup: Web-based systems often have built-in redundancy and backup mechanisms to ensure data integrity and continuity of operations. This minimizes the risk of data loss or system downtime in case of failures.
- Performance monitoring: Web-based systems can offer performance monitoring and analytics tools to track system usage, response times, and overall system health. This data can be used to optimize system performance and identify potential bottlenecks.
- Flexibility and customization: Web-based systems can be easily customized and tailored to meet the specific requirements of an organization. This flexibility allows for the system to adapt to changing business needs and user preferences [58–60].

By taking a system-level approach to understanding web-based systems, organizations can gain a comprehensive understanding of the various components and interactions within the system. This can help in optimizing system performance, enhancing user experience, and achieving business objectives effectively [61].

Overall, these components play crucial roles in facilitating interoperability, data exchange, privacy, coding and classification, standardization, development, and access in EHR systems.

The implementation of EHR indeed comes with its fair share of challenges. To overcome these challenges, several actions can be taken, including adequate planning and requirements assessment, leadership buy-in and support, effective change management, user training and support, standards adoption, use of data privacy and security measures, continuous evaluation and optimization. By addressing these challenges proactively and implementing appropriate strategies, organizations can overcome barriers and leverage the full potential of EHR systems, improving patient care, clinical outcomes, and operational efficiencies [62].

Another important point, in addition to the circuit mentioned above, is recognizing the involvement of civilian healthcare providers in delivering medical services to military personnel. Although military healthcare organizations typically handle primary care, more specialized or advanced treatments are often provided by civilian healthcare facilities. This can complicate the management of military electronic health records (EHRs) since information may need to be shared or integrated between military and civilian healthcare systems. It underscores the importance of seamless coordination and communication between military and civilian healthcare providers to ensure comprehensive and effective healthcare for military personnel. while advanced care is often referred to civilian healthcare providers. This significant dynamic affects the effectiveness and comprehensiveness of military EHRs, as it necessitates a seamless integration of both military and civilian health records to ensure continuity of care for military personnel [63].

## Conclusion

The immediate access to comprehensive patient information, regardless of location, is vital during military deployments and emergency situations to ensure efficient diagnosis, treatment, and monitoring of military personnel. Thus, there is an urgent need to integrate EHR into military healthcare systems [64]. This systematic review contributes to the existing knowledge by providing an overview of EHR usage in military healthcare systems, emphasizing the necessary requirements, available standards, and challenges. It could serve as a strategic starting point for EHR implementation in military healthcare systems, particularly in countries where e-health planning and development are still in early stages. The findings set the stage for future research and provide insight to policymakers and practitioners regarding the potential benefits and obstacles associated with EHR adoption in military healthcare settings. However, the implementation of EHR, especially when dealing with sensitive information, is a time-consuming process that requires a large, multidisciplinary expert team.

### Limitations

The broad subject of EHR includes various aspects and recent studies in this field have a large scope. Therefore, this study focuses mainly on informatics and management issues of EHR implementation in military healthcare systems. However, technical, legal, and organizational issues, which are important bottlenecks, were not covered. It is recommended that future studies address each of these areas separately. Additionally, the study was limited by lack of access to some articles due to unavailable full text and non-English language.

## Supporting information

S1 File PRISMA 2020 checklist. (DOCX)

S2 File Numbered table of all 6051 studies. (XLS)

## Abbreviations

EHR: Electronic Health Record
CMS: Centers for Medicare & Medicaid Services
HL7 FHIR: Health Level Seven Fast Healthcare Interoperability Resources
SQL: Structured Query Language
CCD: Continuity of Care Document
CCR: Continuity of Care Record
SOAP: Simple Object Access Protocol
API: Application Programming Interfaces
SDK: Software Development Kits
HIPAA: Health Insurance Portability and Accountability Act
XML: Extensible Markup Language
HTML: Hyper Text Markup Language
CDA: Clinical Document Architecture
PHR: Personal Health Record
ICD: International Classification of Disease
RAM: Random-Access Memory
CPU: Central Process unit
AHIMA: American Health Information Management Association
SOA: Service-Oriented Architecture
HITECH: Health Information Technology for Economic and Clinical Health
REST: Representational State Transfer
IHE XDS: Integrating the Healthcare Enterprise Cross-Enterprise Document Sharing
WSDL: Web Services Description Language
EMR: Electronic Medical Records
ISO\TC: International Standardization Organization\Technical Committee
SNOMED CT: Systematized Medical Nomenclature for Medicine–Clinical Terminology
DSM: Diagnostic and Statistical Manual of Mental Disorders
OPCS: Office of Population Censuses and Surveys Classification of Interventions and Procedures
NLP: Natural Language Processing
CPOE: Computerized Provider Order Entry
CDR: Clinical Data Repository
PRISMA: Preferred Reporting Items for Systematic Reviews and Meta-analyses
IEEE: Institute of Electrical and Electronics Engineers
SID: Scientific Information Database
ISC: Islamic World Science Citation Center
QUADAS: Quality Assessment of Diagnostic Accuracy Studies
SDK: Software Development Kit
CDSS: Clinical Decision Support System
DoD: Department of Defense
